# A TP53-Pathway-Based Prognostic Signature for Radiotherapy and Functional Validation of TP53I3 in Non-Small-Cell Lung Cancer

**DOI:** 10.3390/cancers18030457

**Published:** 2026-01-30

**Authors:** Xiang Huang, Li Jiao, Xu Cheng, Yue Fang, Jian Qi, Zongtao Hu, Bo Hong, Jinfu Nie, Hongzhi Wang

**Affiliations:** 1 University of Science and Technology of China, Hefei 230052, China; hx168010@mail.ustc.edu.cn; 2 Hefei Cancer Hospital of CAS, Institute of Health and Medical Technology, Hefei Institutes of Physical Science, Chinese Academy of Sciences (CAS), Hefei 230031, Chinajeffnie@cmpt.ac.cn (J.N.)

**Keywords:** non-small-cell lung cancer, TP53 signaling pathway, nomograms, radiotherapy, prognosis, TP53I3

## Abstract

**Background**: Radiation therapy is an important treatment method for non-small-cell lung cancer (NSCLC). However, predicting patient prognosis remains challenging due to considerable interpatient heterogeneity. The TP53 signaling pathway, implicated in tumor radiosensitivity and treatment outcomes, represents a promising predictive biomarker. Accordingly, in this study, we aimed to identify TP53-signaling pathway-related genes and develop a novel prognostic model for risk stratification for NSCLC patients undergoing radiation therapy. **Methods**: Publicly available NSCLC transcriptomic datasets were obtained from the GEO and TCGA databases. Utilizing bioinformatics approaches, we identified differentially expressed genes (DEGs) associated with the TP53 signaling pathway. Feature selection was performed using LASSO regression, followed by the construction of a multivariate-Cox-regression-based prognostic prediction model. In vitro validation was performed using a cell viability assay, colony formation, cell cycle analysis, apoptosis detection, *γ*H2AX immunofluorescence staining and comet electrophoresis. In vivo validation was performed utilizing a subcutaneous tumor-bearing mouse model, where radiosensitivity was assessed by monitoring tumor volume post-irradiation. **Results**: We constructed a robust prognostic prediction model based on five TP53-signaling-pathway-related genes (MDM2, THBS1, TP53I3, ATM, and SESN3), achieving a 5-year AUC of 0.828 in the training set and a 3-year AUC of 0.824 in the validation set. The model exhibited a significant ability to stratify patients into distinct high- and low-risk groups, demonstrating good predictive performance. The poor prognosis observed in the high-risk group was associated with lower infiltration of anti-tumor immune cells but higher infiltration of immunosuppressive cells. Both in vitro and in vivo experiments demonstrated that TP53I3 knockdown significantly enhanced the radiosensitivity of NSCLC through increased DNA damage, cell cycle arrest and apoptosis. **Conclusions**: In this study, a five-gene signature derived from the TP53 signaling pathway was developed, and the model was shown to effectively predict the prognoses of NSCLC patients undergoing radiotherapy. This signature has the potential to be developed into a clinically applicable tool for personalizing radiotherapy regimens for NSCLC.

## Simple Summary

Radiotherapy is a common treatment for non-small cell lung cancer (NSCLC); however, the variability in therapeutic response poses a significant clinical challenge. This study develops a five-gene prognostic model (MDM2, THBS1, TP53I3, ATM, SESN3) from the TP53 signaling pathway to predict outcomes for NSCLC patients receiving radiotherapy. The model demonstrates high accuracy in stratifying patients into distinct risk groups, correlating with specific immune cell infiltration patterns. Experimental validation, both in vitro and in vivo, demonstrates that knockdown of the key gene TP53I3 significantly enhances radiosensitivity. This effect is mediated through increased DNA damage, induction of cell cycle arrest, and promotion of apoptosis. The findings position this gene signature as a promising biomarker tool for personalizing and optimizing radiotherapy regimens in NSCLC.

## 1. Introduction

Globally, lung cancer has long exhibited the highest incidence and mortality among all cancers, posing a serious threat to human life and health [1]. In particular, non-small-cell lung cancer (NSCLC) accounts for more than 80% of lung cancer cases [2,3]. Radiotherapy plays a crucial role in NSCLC treatment, and approximately 60–70% of NSCLC patients undergo radiotherapy [4]. Although it is one of the most effective treatments for NSCLC patients, the 5-year survival rate after radiotherapy varies significantly (about 15–40%) [5]. Many patients exhibit radioresistance during treatment, which is the main cause of poor prognosis and treatment failure among NSCLC patients, directly leading to tumor recurrence and metastasis [6]. There is an urgent need to improve radiotherapy efficacy and reduce the local recurrence rate for precise individualized treatment in NSCLC [7]. However, conventional prognostic indicators (such as TNM staging, performance status score and serum tumor markers) inadequately represent tumor biology; instead, a gene-expression-based molecular prognostic model could supplement clinical decision-making [8].

TP53, an important tumor suppressor gene known as the “guardian of the genome”, encodes the p53 protein, which plays a critical role in a tumor’s response to radiotherapy by regulating signaling pathways for cell cycle arrest, DNA repair, apoptosis and cellular senescence, for example [9]. Upon exposure to ionizing radiation, the wild-type p53 protein initiates cell cycle arrest, DNA damage repair, or programmed cell death by regulating downstream target gene expression, thereby eliminating cells with severely damaged genomes [10]. The mutation rate of TP53 in NSCLC is as high as 50%, and abnormalities in its pathways are closely associated with radiotherapy resistance [11,12]. The expression levels of TP53-signaling-pathway-related genes (such as MDM2, p21, and BAX) can affect the sensitivity of tumor cells to radiotherapy, making them potential prognostic markers [13]. However, to the best of our knowledge, there are few studies on using p53-signaling-pathway-related genes to construct a prognostic model for radiotherapy in NSCLC. Therefore, in this study, we aimed to use bioinformatics techniques to develop a radiotherapy prognostic model for NSCLC based on TP53-signaling-pathway-related genes.

## 2. Materials and Methods

### 2.1. Data Acquirement

The dataset GSE20549 was downloaded from the GEO database (https://www.ncbi.nlm.nih.gov/geo/, accessed on 5 December 2021), which contains microarray data of two NSCLC cell lines (radiation-sensitive H460 and radiation-resistant H1299). The TCGA-NSCLC data was downloaded from the UCSC Xena database (http://xena.ucsc.edu/, accessed on 5 December 2021), including transcriptome data, mutation data, and clinical information for 105 NSCLC samples (Table 1). For the validation dataset, tumor tissue samples from 14 NSCLC patients treated with radiotherapy, including clinical and survival information, were obtained from the biological sample bank of Shanxi Cancer Hospital. The 14 tumor tissue samples were sequenced by RNA-seq. The portions of this study involving biospecimens have been reviewed and approved by the Ethics Committee of Hefei Cancer Hospital, Chinese Academy of Sciences (Approval No.: PJ-KY2025-035).

### 2.2. Screening of Radiosensitivity-Related Genes and Enrichment Analysis of Differential Pathways

The limma package was used to analyze differentially expressed genes between the radiation-sensitive H460 and the radiation-resistant H1299 NSCLC cell lines. The R clusterProfiler package was then employed to perform enrichment analysis of signaling pathways and biological functions for these differentially expressed genes.

### 2.3. Construction and Validation of Prognostic Models

LASSO regression was performed using the glmnet package. LASSO regression and 10-fold cross-validation were used to screen six TP53-associated genes, yielding a total of five key genes. A multivariate Cox analysis was then used to calculate the hazard ratios of these five key genes and construct a prognostic model. Risk scores for each patient were calculated using a risk formula based on gene expression levels and their corresponding weights. Samples were divided into high- and low-risk groups according to the median risk score, and the survival package was employed to compare prognostic differences between them. The timeROC package was used to calculate the area under the curve (AUC) for evaluating the accuracy of the prognostic model. Independent risk factors (risk scores and tumor-bearing status) were screened out using the multivariate Cox analysis, and then, a nomogram was created using the nomogram function in the rms package. A calibration curve was also plotted to validate the discrepancy between our model and the observed patient survival rates.

### 2.4. Functional Enrichment Analysis (GSEA)

To explore the differences in biological functions between the high- and low-risk groups, we downloaded KEGG gene sets from the MSigDB database (http://www.gsea-msigdb.org/gsea/downloads.jsp, accessed on 15 December 2021) and performed GSEA (Gene Set Enrichment Analysis) analysis using the R clusterProfiler package. The ssGSEA algorithm from the GSVA package and xCell were used to analyze the transcriptome data of tumor samples, infer the relative enrichment scores of immune-infiltrating cells, and compare the differences in immune cell infiltration levels between high- and low-risk score groups. The Tumor Immune Dysfunction and Exclusion (TIDE) algorithm was used to predict patients’ response to immunotherapy. TIDE scores for each sample were calculated using the TIDE database (http://tide.dfci.harvard.edu/, accessed on 15 December 2021).

### 2.5. Transcriptome Sequencing of NSCLC Tissue Samples

According to the manufacturer’s protocol, RNA isolation and purification from NSCLC tissue samples were performed using an RNA extraction kit (TransGen Biotech, Beijing, China). Qualitative and quantitative tests were then conducted via a Nanodrop (Thermo Scientific, Waltham, MA, USA). After the library was constructed using the NEBNext Ultra II RNA Library Prep Kit for Illumina (NEB, Ipswich, MA, USA), RNA sequencing was performed on the NovaSeq6000 platform using paired-end sequencing. Raw sequencing data were quality-controlled using trim-galore and mapped to the human hg19 reference genome using STAR software (v2.7.10b), and gene expression levels were quantified according to transcripts per million (TPM) using RSEM software (v1.3.1).

### 2.6. Cell Culture and Irradiation Treatment

The NSCLC A549, H460 and H1299 cell lines were cultured in RPMI-1640 medium (Corning, NY, USA) containing 10% fetal bovine serum (ExCell Bio, Shanghai, China) and 1% penicillin–streptomycin solution (HyClone, Logan, UT, USA). Human normal lung epithelial cell line BEAS-2B was cultured in complete DMEM medium (Corning, NY, USA). All cells were routinely maintained in a constant-temperature sterile incubator at 37 °C with 5% CO_2_.

The irradiation treatment for all cell lines was as follows: 6MV X-rays, source–skin distance (SSD) irradiation at 100 cm, and a dose rate of 300 cGy/min (Elekta Medical Linear Accelerator, Infinity, Swedenthe). Irradiation doses were 0, 2, 4, 6, and 8 Gy respectively, according to experimental requirements.

### 2.7. Establishment of NSCLC Cell Line with Stable Knockdown of TP53I3 Gene

Lentiviruses with non-targeting shRNA control and TP53I3 shRNA were produced by GenePharma (Shanghai, China).The shRNA sequence for TP53I3 was as follows: shTP53I3: 5′-GCAGCTGCTGGATTCAATTAC-3′.

NSCLC cells were seeded in 6-well plates at 1 × 10^5^ cells/well and incubated with viral supernatant diluted in complete medium containing 5 μg/mL polybrene. After 24 h, the medium was replaced with fresh complete medium. Transduced cells were selected with 5 μg/mL puromycin for 7 days, and stable pools were expanded. The knockdown of TP53I3 expression was validated by Western blot and qRT-PCR.

### 2.8. Western Blot

Total cellular proteins were extracted using RIPA lysis buffer (Beyotime, Shanghai, China) supplemented with 1× protease inhibitor cocktail (Sparkjade, Jinan, China). Cell lysates were centrifuged at 13,300 r/min for 10 min at 4 °C, and the supernatant was collected. Protein concentration was determined using a BCA Protein Assay Kit (Beyotime, Shanghai, China) according to the manufacturer’s instructions. The samples were then subjected to SDS-PAGE, and proteins were then transferred to a PVDF membrane (Millipore, Darmstadt, Germany) using a wet transfer system at 100 V for 60 min. The membrane was blocked with 10% non-fat dry milk in TBST for 2 h at room temperature, followed by incubation with primary TP53I3 antibody (14828-1-AP, Proteintech, Wuhan, China) and *β*-actin antibody (HC201, TransGen Biotech, Beijing, China) overnight at 4 °C. On the following day, the membranes were washed with TBST and incubated with species-specific HRP-conjugated secondary antibodies (CST, Danvers, MA, USA) for 1 h. Following washing, protein bands were visualized using an enhanced chemiluminescence (ECL) kit (NCM Biotech, Suzhou, China) and detected with a ChemiDoc XRS+ imaging system (PeiQing, Shanghai, China).

### 2.9. Real-Time PCR

Total RNA was extracted using the RNA Extraction Kit (TIANGEN, Beijing, China), and the RNA concentration was determined using a spectrophotometer (DS-11, Denovix, Wilmington, DE, USA). Reverse transcription was performed using the Script Reverse Transcription Supermix Kit (TransGen Biotech, Beijing, China) to generate cDNA. Real-time PCR was carried out using the Power SYBR Green PCR Master Mix (Invitrogen, Carlsbad, CA, USA) on a Real-time Quantitative PCR Instrument (HealForce, Hangzhou, China). The results were analyzed using the 2^−ΔΔCT^ method, with data normalized to *β*-actin. The primers utilized in this study were as follows:

TP53I3 Forward: 5′-CTGGAGCTATTCCTCTGGTCAC-3′;
TP53I3 Reverse: 5′-GTTCTTCTCCCAGTAGGATCCG-3′;
*β*-actin Forward: 5′-CATGTACGTTGCTATCCAGGC-3′;
*β*-actin Reverse: 5′-CTCCTTAATGTCACGCACGAT-3′.

### 2.10. Colony Formation and Cell Viability Assays

For the colony formation assay, 500 cells per well were plated on 6-well plates. After 24 h, the cells were irradiated under 0, 2, 4, 6 and 8 Gy doses. After 10–14 days, the colonies were fixed using methanol and stained for 30 min with 0.1% crystal violet. Clones were counted using Image J software (NIH, Bethesda, MD, USA). For the cell viability assay, NSCLC cells were seeded into 96-well plates at a density of 5 × 10^3^ cells per well. After 24 h, cells were irradiated at doses of 0, 2, 4, 6 and 8 Gy, respectively, and the culture was continued. After 24 h, according to the instructions of the Cell Titer-Glo Cell Viability Assay Kit (Promega, Beijing, China), 20 μL of CTG reagent was added to each well, and the plates were incubated at 37 °C in the dark for 10 min. The luminescence signal of each well was read using a microplate reader (LumiStation 1800, Flash, Shanghai, China).

### 2.11. Cell Cycle and Apoptosis Assay

The cell cycle was evaluated with the PI/RNase staining kit (BD Bio sciences, San Diego, CA, USA). At 48 h post-irradiation, cells were collected and fixed in precooled 75% ethanol at −20 °C for 24 h, followed by washing twice with phosphate-buffered saline (PBS) and staining with PI/RNase staining buffer for 15 min. The stained cells were counted using a flow cytometer (Beckman Coulter, Brea, CA, USA).

Apoptosis analysis was performed with an FITC Annexin V apoptosis detection kit (BD Bio sciences, San Diego, CA, USA) according to the manufacturer’s instructions. Cells were detached with EDTA-free trypsin, and the cell pellet was collected by centrifugation. The pellet was washed once with PBS and resuspended in 500 μL of 1× binding buffer. Then, 100 μL of the cell suspension was transferred to a flow cytometry tube. First, 5 μL of FITC dye was added, and the mixture was incubated in the dark for 15 min. Subsequently, 5 μL of PI dye was added, followed by dark incubation for 5 min. After staining, 400 μL of 1× binding buffer was added to each tube, and apoptosis was detected by flow cytometry (Beckman Coulter, Brea, CA, USA).

### 2.12. Comet Assay

The comet assay was performed to assess DNA damage in the cells using a comet assay kit (Beyotime, Shanghai, China) and following the manufacturer’s instructions. The cells were detached with trypsin, and the cell pellet was collected by centrifugation. The pellet was washed once with pre-cooled PBS, resuspended in PBS to a cell density of 1 × 10^6^ cells/mL. Then, 1% agarose gel (normal melting point) was used to prepare the first layer of gel. After, 10 µL of cells (approximately 10^4^ cells) were mixed with 75 μL of 0.7% agarose (low melting point) pre-warmed in a 37 °C water bath. Then, 70 μL of the mixture was quickly pipetted onto the first gel layer, covered with a coverslip, and stored at 4 °C for 10 min to solidify the second gel layer. The slides were lysed in lysis buffer (Lysis Buffer:DMSO = 9:1) at 4 °C for 1–2 h, then rinsed with PBS for 3 min. The slides were placed in a horizontal electrophoresis tank, and electrophoresis buffer was poured to a level at least 0.25 cm above the gel surface. The tank was left at room temperature for 20–60 min to allow DNA unwinding. Electrophoresis was performed at a low voltage of 25 V for 20–30 min. After electrophoresis, the slides were placed in a Petri dish and neutralized with neutral buffer at 4 °C 1–3 times for 5–10 min each. The neutral buffer was discarded, and approximately 20 μL of PI solution was added to the slides for staining in the dark for 10–20 min. The slides were then washed three times with ultrapure water, covered with coverslips, and observed under a fluorescence microscope (ZEISS, Oberkochen, Germany).

### 2.13. Immunofluorescent Staining

Cell slides were first placed in 12-well plates, followed by cell seeding. Once the cells adhered to the slides, they were exposed to 8 Gy of radiation. The cells were then continuously cultured for an additional 12 h. The cells were rinsed three times with PBS, fixed with 4% paraformaldehyde at 37 °C for 20 min, and then permeabilized with a PBS solution containing 0.1% Triton X-100 for 10 min. The cells were then incubated with the primary *γ*H2AX antibody (2577S, CST, Danvers, MA, USA) at 4 °C overnight. After three washes with PBS, the secondary antibody conjugated to Alexa Fluor 488 (A21206, Invitrogen, Carlsbad, CA, USA) was applied in the dark for 40 min at room temperature. Nuclei were counterstained with 1 μg/mL DAPI (Solarbio, Beijing, China) for 5 min, and the samples were mounted with antifade mounting medium before visualization using a fluorescence microscope (ZEISS, Germany).

### 2.14. Xenograft Tumor Experiment

Female BALB/c nude mice aged 4–6 weeks were raised at the Experimental Animal Center, Hefei Cancer Hospital, Chinese Academy of Sciences. A dose of 5 × 10^6^ A549 cells with stable transfection of control shRNA and TP53I3 shRNA mixed with Matrigel (Corning, NY, USA) were inoculated subcutaneously into the axillary region of the right forelimb of mice. When the average tumor volume reached 100–150 mm^3^, the mice were randomly divided into 4 groups: NC, NC + IR, sh-TP53I3 and sh-TP53I3 + IR (n = 10 per group). The irradiation group received a total dose of 10 Gy from an Elekta medical linear accelerator targeting the tumors. The maximum and minimum diameters of the tumors were measured with a caliper every three days to calculate the tumor volume using the formula (mm^3^) = (L × W^2^)/2, where L and W represent the major and minor diameters of the tumor, respectively. At the end of the experiment, the mice were euthanized by CO_2_ asphyxiation. Tumor tissues were dissected, photographed, and fixed in 10% formalin. All animal experimental procedures were conducted in accordance with a protocol (Approval No.: DWLLPF-2024062501) approved by the Experimental Animal Ethics Committee of Hefei Cancer Hospital, Chinese Academy of Sciences.

### 2.15. Statistical Analysis

All data analyses were performed using R4.1.3 software and Rstudio software (v1.4.1717). Unless otherwise specified, the Wilcoxon rank-sum test and *t* test were used to compare continuous variables between two groups, and the Kruskal test was used to compare continuous variables across multiple groups. Survival analysis was conducted using Kaplan–Meier curve analysis. *p* < 0.05 was considered statistically significant (*p* < 0.05 *, *p* < 0.01 **, *p* < 0.001 *** and *p* < 0.0001 ****).

## 3. Results

### 3.1. Analysis of Radiosensitivity-Related Genes in NSCLC

**Figure 1:**
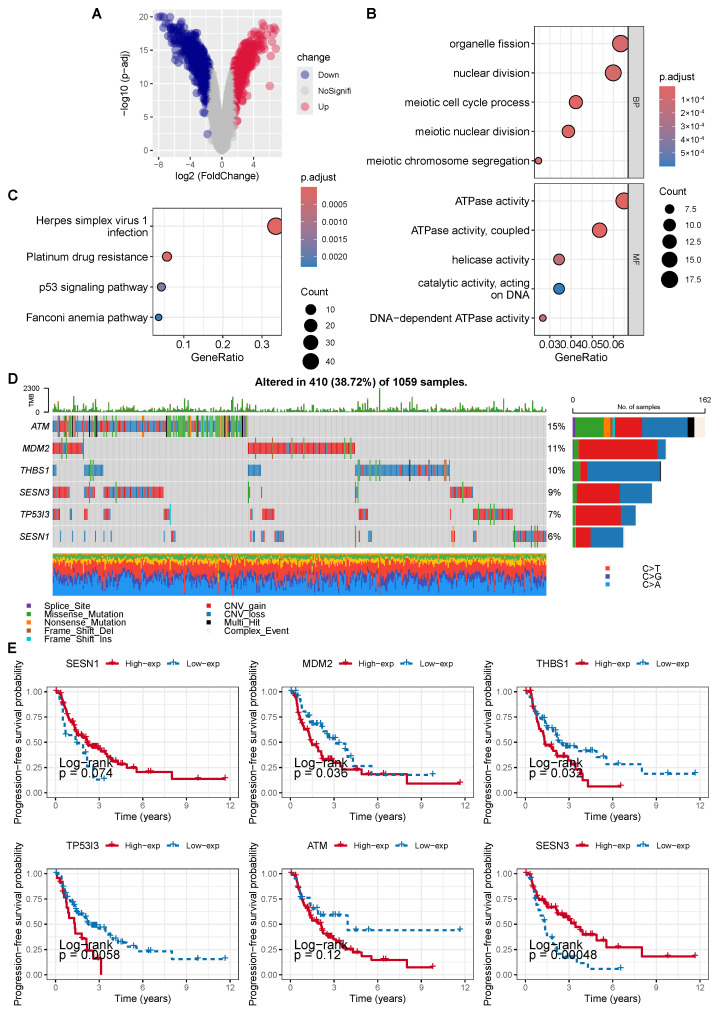
(**A**) Volcano plot of differentially expressed genes between the radiosensitive group and the radioresistant group. (**B**) GO pathway enrichment analysis of differentially expressed genes. (**C**) KEGG pathway enrichment analysis of differentially expressed genes. (**D**) Waterfall plot showing the mutation and CNV status of 6 genes involved in the p53 pathway. The upper bar chart indicates the number of gene mutations in each sample. The bottom annotations with different colors represent various mutation types in the each sample. (**E**) Kaplan–Meier curves indicating the relationship between the expression of the 6 genes and the progression-free survival of NSCLC patients. A log-rank test *p* < 0.05 was considered statistically significant.

To identify radiosensitivity-related genes in NSCLC, we performed differential gene expression analysis between the radiosensitive and radioresistant cell lines H460 and H1299, respectively. Using a threshold of an adjusted *p* value < 0.05 and |log2 FC| > 1.5, a total of 1354 differentially expressed genes (DEGs) were identified, including 611 up-regulated genes and 743 down-regulated genes (Figure 1A). To investigate the functions and signaling pathways of these genes, we conducted GO and KEGG enrichment analyses on the DEGs. GO analysis showed that the DEGs were primarily involved in organelle fission, nuclear division and the regulation of ATPase activity (Figure 1B). The KEGG pathway analysis revealed that the DEGs were predominantly enriched in pathways including platinum drug resistance and the TP53 signaling pathway (Figure 1C). Among these DEGs, six genes were enriched in the TP53 signaling pathway, including SESN1, MDM2, THBS1, TP53I3, ATM and SESN3.

We analyzed the mutation and copy number variation (CNV) of these six genes in NSCLC using the TCGA database. The results showed that 38.72% of the NSCLC cases had mutations in at least one of these genes, with ATM being the most frequently mutated gene (15% mutation frequency). The CNV frequencies of these six genes were also high, with MDM2 primarily showing CNV amplification and THBS1 primarily showing CNV deletion (Figure 1D).

Subsequently, we performed a survival analysis on these six genes using data on radiotherapy-treated NSCLC from the TCGA database. The results showed that four of the six genes (MDM2, THBS1, TP53I3 and SESN3) were significantly associated with progression-free survival (PFS), and among them, only SESN3 was identified as a protective factor (Figure 1E).

### 3.2. Construction and Validation of a Prognostic Model Based on Radiosensitivity-Related Genes

**Figure 2:**
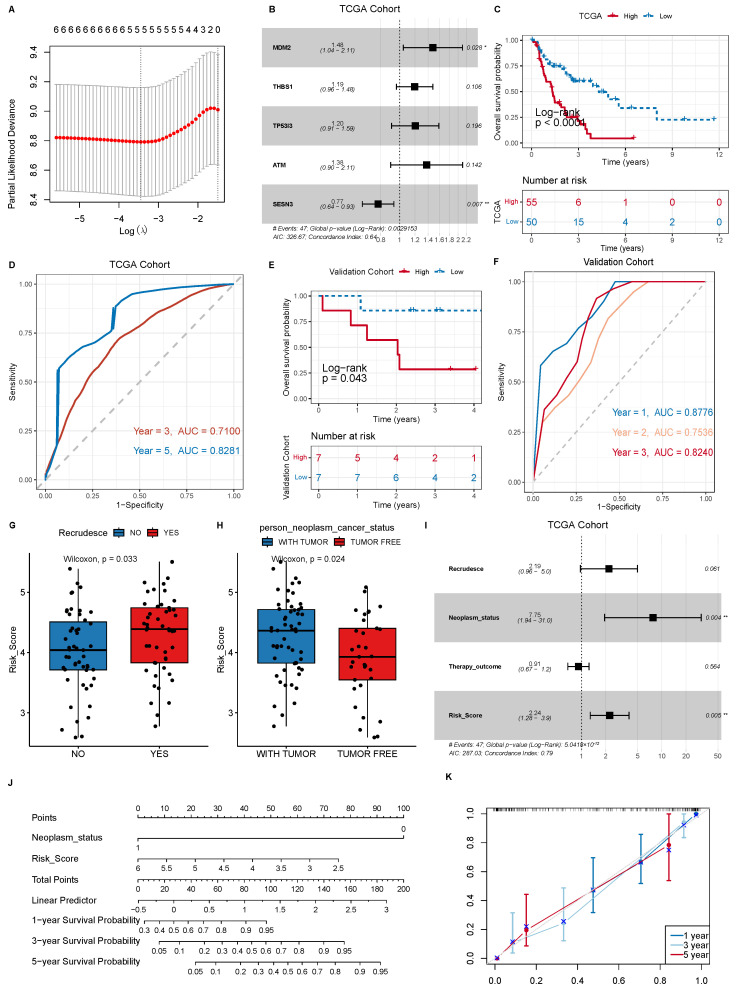
(**A**) LASSO regression analysis was performed to determine the optimal lambda value and its corresponding coefficients. (**B**) The forest plot shows the hazard ratios (HRs) and *p*-values from the multivariate Cox regression analysis of five genes (p<0.05 *, p<0.01 **). (**C**) The Kaplan–Meier survival curve demonstrates that the overall survival of NSCLC patients in the high-risk group was significantly lower than that in the low-risk group in the TCGA dataset. (**D**) Time-dependent ROC curve analysis was conducted to evaluate the risk score for predicting 3- and 5-year prognoses of patients in the TCGA dataset. (**E**) In the validation set, the Kaplan–Meier curve showed that the overall survival of NSCLC patients in the high-risk group was significantly lower than that in the low-risk group. (**F**) Time-dependent ROC curve analysis was performed to assess the risk score for predicting 1-, 2- and 3-year prognoses of patients in the validation set. (**G**) The difference in risk scores between patients with recurrence and those without recurrence. (**H**) The difference in risk scores between patients with different tumor statuses. (**I**) The forest plot shows the hazard ratios (HRs) and P-values from the multivariate Cox regression analysis of the risk score model and clinical traits (p<0.01 **). (**J**) A nomogram model for predicting overall survival in NSCLC patients. (**K**) Calibration curves for the 1-, 2- and 3-year overall survival prediction by the nomogram model.

To obtain hub radiosensitivity-related genes for the prognosis, we conducted a LASSO regression analysis on these six genes and screened out five hub genes (MDM2, THBS1, TP53I3, ATM and SESN3) (Figure 2A). Then, we used a multivariate Cox analysis to construct a model for these five genes. The formula for calculating the model’s risk score is *Risk*_*Score* = −0.394 × (*expression of MDM*2) + 0.177 × (*expression of THBS*1) + 0.185 × (*expression of TP*53*I*3) + 0.320 × (*expression of ATM*) − 0.255 × (*expression of SESN*3) (Figure 2B).

To further evaluate the ability of the model to predict the prognosis, we divided the TCGA NSCLC patients into high- and low-risk groups according to median risk score. The NSCLC patients in the low-risk group showed significant survival benefits. Moreover, the AUCs of the ROC curve of the model for predicting 3- and 5-year prognoses were 0.710 and 0.828, respectively (Figure 2C,D).

Finally, we used an independent cohort to validate the accuracy of the model. Tumor tissue samples from 14 NSCLC patients treated with radiotherapy were obtained from the biological sample bank of Shanxi Cancer Hospital, and then were sequenced by RNA-seq. In the validation cohort, the NSCLC patients in the low-risk group showed significantly better survival (*p* = 0.043), and the AUCs of the time-dependent ROC curve at 1, 2 and 3 years were 0.8776, 0.7536 and 0.824, respectively (Figure 2E,F). These results indicate that the model performs well in predicting the prognosis of NSCLC patients treated with radiotherapy.

We further analyzed the relationship between clinical characteristics and risk scores. The model-predicted risk scores exhibited significant differences with recurrence status and tumor-bearing state (Figure 2G,H). NSCLC patients with recurrence or tumor-bearing survival had higher risk scores. To validate whether the model was independent of other factors, we used univariate Cox analysis to assess the hazard ratios (HRs) of clinical characteristics and model-predicted risk score. The univariate Cox analysis identified three clinical characteristics (tumor-bearing status, recurrence and therapy outcome) and the model risk score as significantly prognostic factors. We then performed a multivariate Cox analysis, and found that only the risk score and tumor-bearing status exhibited significant prognostic value, indicating that the model-predicted risk score was independent of other risk factors (Figure 2I).

To construct a more robust model, we combined the risk score and tumor-bearing status to develop a nomogram model (Figure 2J). The calibration curves for the nomogram at 3 and 5 years were close to the diagonal (Figure 2K), indicating the nomogram’s good predictive ability. Additionally, we calculated the c-index of the nomogram as 0.758, demonstrating the model’s high accuracy.

### 3.3. Clinical and Biological Significance of the Prognostic Model

**Figure 3:**
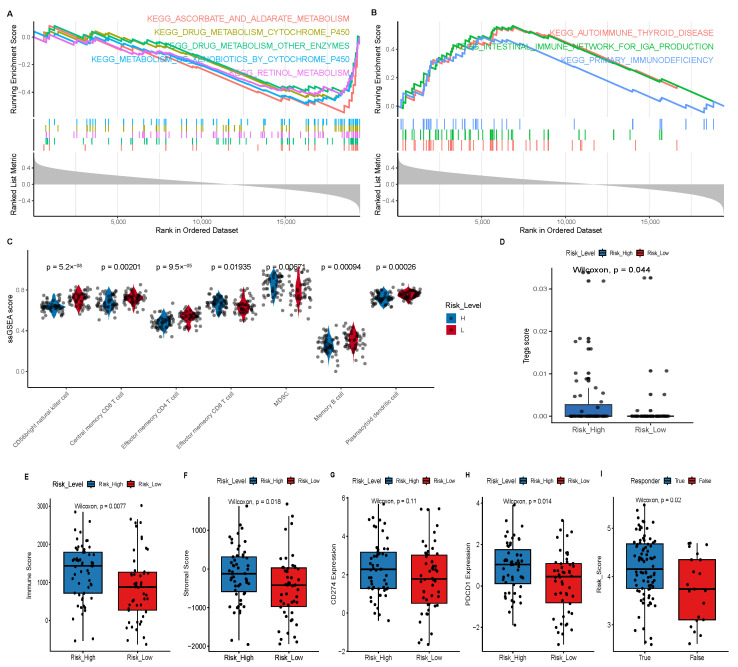
(**A**) GSEA analysis shows that the low-risk group is enriched in metabolism-related pathways. (**B**) GSEA analysis shows that the high-risk group is enriched in immunity-related pathways. (**C**) GSVA analysis shows the differences in immune cell infiltration between the high- and low-risk groups. (**D**) xCell analysis shows the difference in immune scores for Tregs cells between the high- and low-risk groups. (**E**) Comparison of immune scores between high- and low-risk groups. (**F**) Comparison of stromal scores between high- and low- groups. (**G**) Expression of PD-L1 (CD274) in low- and high-risk groups. (**H**) Expression of PD-1 (PDCD1) in low- and high-risk groups. (**I**) The difference in risk scores between the immunotherapy-responsive and non-responsive groups predicted by TIDE.

We analyzed the potential pathways associated with the model-predicted risk score using the GSEA algorithm. The GSEA showed that the risk score was associated with immunity and drug metabolism (Figure 3A,B). Immune-related pathways such as PRIMARY_IMMUNODEFICIENCY, AUTOIMMUNE_THYROID_DISEASE, and INTESTINAL_IMMUNE_NETWORK_FOR_IGA_PRODUCTION were mostly enriched in the high-risk group, while drug metabolism-related pathways such as ASCORBATE_AND_ALDARATE_METABOLISM, DRUG_METABOLISM_OTHER_ENZYMES, and DRUG_METABOLISM_CYTOCHROME_P450 were predominantly enriched in the low-risk group. These findings suggest that the model-predicted risk score may be linked to drug metabolism and immunity.

The GSEA analysis suggested that the model-predicted risk score might be associated with immunity. Thus, we further analyzed the relationship between the risk score and immune infiltration. We used the GSVA algorithm to analyze the infiltration scores of 28 types of immune cells in all 105 NSCLC samples. The results showed that most immune cells, including natural killer cells, central memory CD8+ T cells, effector memory CD4+ T cells and effector memory CD8+ T cells, displayed lower infiltration in the high-risk group; immunosuppressive cells MDSCs exhibited higher infiltration in the high-risk group (Figure 3C). Moreover, xCell algorithm indicated that Tregs cells exhibited higher immune scores in the high-risk group (Figure 3D). We also used the estimate algorithm to analyze the relationship of the risk score with the immune or stromal score. The results showed that both the immune and stromal scores in the high-risk group were significantly higher than those in the low-risk group (Figure 3E,F).

The model-predicted risk score is associated with immune cell infiltration. To further indicate the relationship between the risk score and response to immunotherapy, we firstly analyzed the relationship between the model and PD-1/PD-L1 expression, and found that the expression levels of PD-1 and PD-L1 in the high-risk group were significantly higher than those in the low-risk group, further suggesting that the model may predict responses to immunotherapy (Figure 3G,H). Furthermore, we used the TIDE algorithm to predict the response of each NSCLC sample to immunotherapy; it showed that higher risk scores may be associated with a higher response rate to immunotherapy, indicating that the high-risk group is more likely to respond to immunotherapy (Figure 3I).

### 3.4. Knockdown of TP53I3 Enhances the Sensitivity of NSCLC Cells to Irradiation

We found that five TP53 pathway-related genes (MDM2, THBS1, TP53I3, ATM and SESN3) were associated with the prognosis of NSCLC patients treated with radiotherapy. As a downstream target gene of p53, TP53I3 expression is induced by DNA damage (such as ionizing radiation). TP53I3 functions in the regulation of oxidative stress, cell survival and apoptosis [14]. Therefore, we selected TP53I3 for further functional study.

**Figure 4:**
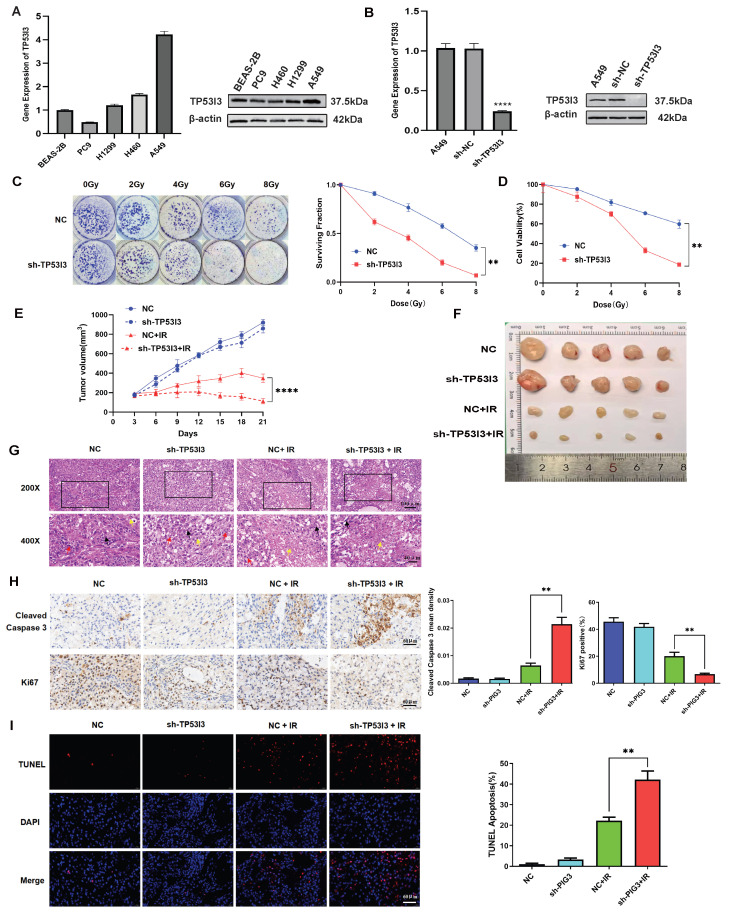
(**A**) TP53I3 mRNA and protein expression levels in BEAS-2B, PC9, H1299, H460 and A549 cells. (**B**) qRT-PCR and Western blot show that TP53I3 is knocked down in A549 cells stably transfected with TP53I3 shRNA (p<0.0001 ****). (**C**) Colony formation analysis in A549 cells with TP53I3 knockdown after 0, 2, 4, 6 and 8 Gy of irradiation (p<0.01 **). (**D**) Cell viability assay in A549 cells with TP53I3 knockdown after 0, 2, 4, 6 and 8 Gy of irradiation (p<0.01 **). (**E**) Growth curve of A549 xenografts in NC and TP53I3 knockdown groups with or without irradiation treatment (p<0.0001 ****). (**F**) Representative images of tumors isolated from tumor-bearing mice at the end of the experiment. (**G**) HE staining in tumor tissues isolated in different groups. (**H**) IHC staining and quantitative analysis of Cleaved Caspase 3 and Ki67 in tumor tissues of different groups (p<0.01 **). (**I**) TUNEL staining in tumor tissues of different groups (p<0.01 **). The uncropped blots are shown in Supplementary Materials.

Firstly, we detected the expression of TP53I3 protein and mRNA in the NSCLC H1299, H460 and A549 cell lines as well as normal lung epithelial cell line BEAS-2B. The result indicated that A549 cells showed the highest TP53I3 mRNA and protein expression levels (Figure 4A). Therefore, to further investigate the effect of TP53I3 on cellular radiosensitivity, we established A549 cells with TP53I3 stably knocked down. The knockdown efficiency was detected by RT-PCR and Western blot (Figure 4B). The colony formation assay showed that radiation reduced the ability of colony formation in A549 cells. When TP53I3 was knocked down, the A549 cells were more sensitive to radiation (Figure 4C). Furthermore, the cell viability assay indicated that TP53I3 knockdown enhanced the inhibition of proliferation by radiation in A549 cells (Figure 4D).

To further explore the role of TP53I3 in vivo, we detected the radiosensitivity of nude mice xenografts established using A549 cells with stable TP53I3 knockdown. When the tumor volume reached 170–200 mm^3^, tumor-bearing mice were subjected to local irradiation, and tumor growth and body weight were measured every 3 days. The result showed that radiation induced more growth inhibition in A549 xenografts with stable TP53I3 knockdown, compared to stable transfection of control shRNA (Figure 4E,F). At the end of the experiment, we dissected the tumors from the mice for histological analysis. HE staining showed that radiation induced massive tumor cell necrosis, nuclear fragmentation or disappearance, and reduced nuclear mitotic figures in the control shRNA group. In the TP53I3 shRNA group, radiation-induced tumor necrosis was increased (Figure 4G). Immunohistochemical staining showed that radiation induced stronger expression of Cleaved Caspase 3, and inhibited stronger expression of Ki67 in the TP53I3 shRNA group, compared to the control shRNA group (Figure 4H). The TUNEL assay showed that radiation induced more apoptosis in the TP53I3 shRNA group compared to the control shRNA group (Figure 4I). Therefore, these results suggest that TP53I3 knockdown enhances the radiosensitivity of NSCLC in vitro and in vivo.

### 3.5. Knockdown of TP53I3 Enhances Radiation-Induced DNA Damage, Cell Cycle Arrest and Apoptosis in NSCLC Cells

**Figure 5:**
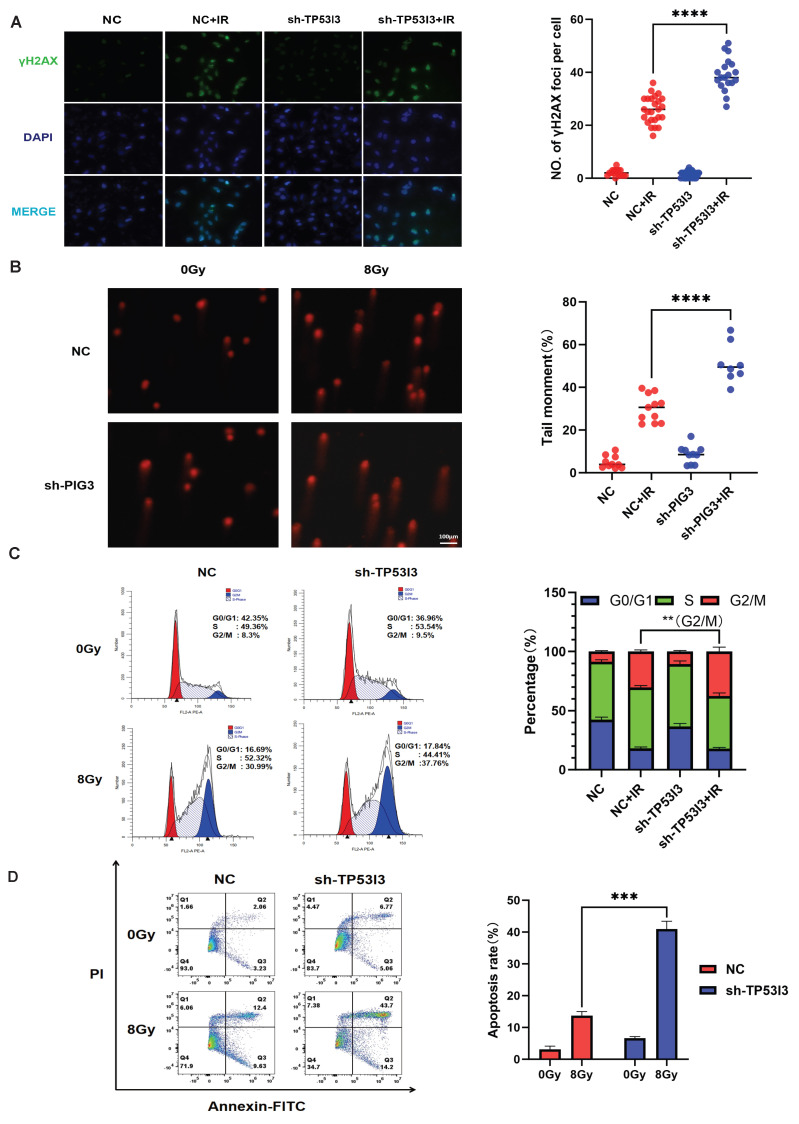
(**A**) Immunofluorescent staining and quantitative analysis of *γ*H2AX foci in A549 cells stably transfected with control and TP53I3 shRNA at 12 h post-irradiation at 8 Gy (p<0.0001 ****). (**B**) Representative images and quantitative analysis of comet assay in A549 cells stably transfected with control and TP53I3 shRNA at 12 h post-irradiation at 8 Gy (p<0.0001 ****). (**C**) Cell cycle analysis of A549 cells stably transfected with control and TP53I3 shRNA at 12 h post-irradiation at 8 Gy (p<0.01 **). (**D**) Apoptosis of A549 cells stably transfected with control and TP53I3 shRNA, as detected at 48 h post-irradiation at 8 Gy (p<0.001 ***).

The radioresistant phenotype is commonly associated with DNA damage repair. To investigate the impact of TP53I3 on DNA damage repair, we performed immunofluorescence assays to detect *γ*H2AX expression. As a marker of DNA double-strand breaks, radiation-induced *γ*H2AX foci were significantly higher in the TP53I3-knockdown A549 cells than in the control cells (Figure 5A). Similarly, according to the comet assay, TP53I3 knockdown resulted in a significant increase in the comet tail moment at 12 h post-IR (8 Gy) (Figure 5B).

We further analyzed radiation-induced cell cycle arrest and apoptosis when TP53I3 was knocked down in A549 cells. The result indicated that radiation induced more G2/M phase arrest in A549 cells with stable TP53I3 knockdown, compared with A549 cells with stable control shRNA transfection (Figure 5C). Consistently, increased radiation-induced apoptosis was also observed in A549 cells with stable TP53I3 knockdown (Figure 5D). Therefore, TP53I3 knockdown enhances radiation-induced DNA damage, G2/M cell cycle arrest and cell apoptosis in NSCLC cells.

## 4. Discussion

In this study, we employed bioinformatics approaches to analyze differentially expressed genes between radio-sensitive and radio-resistant NSCLC cell lines. Through GO and KEGG analysis, six differentially expressed genes (SESN1, MDM2, THBS1, TP53I3, ATM and SESN3) were significantly enriched in the TP53 signaling pathway. Then, a LASSO regression analysis screened out five hub genes (MDM2, THBS1, TP53I3, ATM and SESN3). Using the five genes, by multivariate Cox analysis, we constructed a prognostic model, which displayed good predictive performance in the NSCLC training and validation set. Finally, we functionally validated that TP53I3 knockdown enhanced radiation-induced cell death in NSCLC in both in vitro and xenograft models.

As a core regulator of genomic stability, TP53 plays a pivotal role in mediating tumor cells’ responses to radiotherapy [15,16]. Abnormalities in the p53 pathway drive radiotherapy resistance in tumor cells through three major pathways: defective DNA damage repair, impaired apoptotic signaling, and remodeling of the tumor microenvironment [17,18,19]. These five key genes from the prognostic model may modulate radiotherapy sensitivity in NSCLC through multiple mechanisms and pathways. By mediating p53 ubiquitination and degradation, MDM2 acts as its key upstream negative regulator to inhibit radiotherapy-induced apoptosis [20]. Meanwhile, MDM2 can activate the PI3K/AKT signaling pathway to enhance DNA damage repair capacity [21]. Clinical studies have shown that MDM2 gene amplification leads to persistent inhibition of p53 activity, which is significantly associated with radiotherapy resistance in tumors [22]. THBS1, a p53-regulated angiogenesis inhibitor, activates the TGF-*β*/Smad3 pathway to promote extracellular matrix remodeling and inhibit radiotherapy-induced DNA damage repair [23]. THBS1 serves as a bridge between radiation damage response and tumor microenvironment regulation, giving it dual effects on radiotherapy efficacy. While THBS1 enhances radiosensitivity by inhibiting abnormal angiogenesis, it also promotes a radioresistant phenotype by activating fibrotic signals and immunosuppressive networks [24]. ATM acts as a central signaling hub in the DNA damage response (DDR) [25]. Ionizing radiation-induced DNA double-strand breaks activate ATM via the MRN complex, triggering its dimer dissociation and autophosphorylation at S1981. Once activated, the ATM-Chk2 axis phosphorylates p53 at S15/S20, leading to coordinated cell cycle arrest at G1/S and G2/M checkpoints or initiation of mitochondrial apoptosis, thereby forming the core DDR regulatory network [26]. Dysfunction in this pathway, including reduced HR repair efficiency or checkpoint failure, contributes to heterogeneity in tumor cell radiosensitivity [27]. SESN3 (sestrin 3), a p53-activated stress-responsive protein of the sestrin family, inhibits autophagy and exacerbates radiotherapy-induced oxidative stress by activating the AMPK/mTORC1 pathway [28]. SESN3 acts as a central integrator of energy metabolism, oxidative stress, and autophagy-apoptosis balance, thereby linking p53 signaling to radiotherapy sensitivity [29].

TP53I3 (Tumor Protein P53 Inducible Protein 3), a p53-inducible gene, is homologous to oxidoreductases and modulates reactive oxygen species (ROS) synthesis and oxidative stress [14,30]. TP53I3 blocks apoptotic signaling by downregulating pro-apoptotic proteins (e.g., Bax) and upregulating anti-apoptotic proteins (e.g., Bcl-2) [31]. Moreover, TP53I3 reduces ROS levels by enhancing antioxidant enzyme activity (e.g., SOD and CAT), thereby inhibiting apoptosis [32]. TP53I3 also plays a key role in the DNA damage response (DDR) by upregulating repair proteins (e.g., ATM, BRCA1) and activating pathways like NHEJ and HR, thereby accelerating DNA repair and reducing cell death [33]. Downregulation of TP53I3 expression reduces Chk1/Chk2 phosphorylation following DNA damage, which impairs the S and G2/M phase checkpoints and ultimately prevents effective DNA repair [34]. By reducing H2AX phosphorylation, TP53I3 silencing impairs the DNA damage response. This increases cellular susceptibility to agents like UV and chemotherapy, leading to reduced survival [35]. TP53I3 acts as an oncogene in lung adenocarcinoma cells by activating the PI3K/AKT/mTOR signaling pathway [36]. In this study, we find that high TP53I3 expression is associated with poor survival and radiotherapy resistance in NSCLC. We functionally validate that TP53I3 knockdown sensitizes NSCLC cells to radiation by weakened DNA damage repair capacity, thereby enhancing radiation-induced G2/M phase arrest and apoptosis. Thereby, these findings position TP53I3 as a promising target and biomarker for improving radiotherapy in NSCLC.

Based on the above five core genes, we constructed a prognostic prediction model using a multivariate Cox proportional hazards model. The GSEA analysis revealed that the high-risk group of this prognostic model was significantly enriched in immune-related signaling pathways. Among them, the PRIMARY IMMUNODEFICIENCY pathway reflects congenital immune dysfunction, including defects in T/B lymphocyte development, antigen recognition signaling, and innate immune effector functions. In NSCLC, enrichment of this pathway may indicate T cell functional exhaustion, which is associated with high PD-L1 expression and immunotherapy resistance [37,38]. To analyze the association between the model and the immune microenvironment, we quantified the infiltration scores of immune cells. The results showed lower infiltration of anti-tumor immune cells but higher infiltration of immunosuppressive cells (e.g., MDSCs, Tregs) in the high-risk group. The TIDE algorithm showed that the high-risk group had elevated immunotherapy-response scores, suggesting a possibly increased sensitivity to PD-1/PD-L1 blockade. This implies that these patients may benefit from a combination of radiotherapy and immunotherapy. Radiotherapy can induce immunogenic cell death to release tumor antigens, thereby synergizing with PD-1/PD-L1 inhibitors to alleviate immunosuppression and enhance tumor killing by cytotoxic T lymphocytes [39]. These findings provide a rationale for combining radiotherapy with immunotherapy in high-risk patients to enhance treatment efficacy.

This study also has several limitations. Firstly, the prognostic model was developed using data from 105 radiotherapy-treated NSCLC patients in the TCGA database and then underwent exploratory validation in an independent cohort of 14 patients. This limited external validation cohort size raises concerns about potential model overfitting. We propose that future work quantify the mRNA levels of the five genes (MDM2, THBS1, TP53I3, ATM, and SESN3) by PCR, thereby validating the prognostic model in a larger NSCLC cohort. Secondly, the prediction of immunotherapy response is currently limited to the TIDE algorithm. Consequently, future studies will investigate whether model-predicted high-risk NSCLC patients actually benefit from immunotherapy in a clinically relevant cohort receiving immunotherapy. Thirdly, given the small sample size, we combined NSCLC patients with adenocarcinoma and squamous cell carcinoma into one group for analysis. Lung adenocarcinoma and squamous cell carcinoma exhibit marked differences in histology, molecular characteristics, and response to radiotherapy. Therefore, the model’s efficacy needs to be further investigated in lung adenocarcinoma and squamous cell carcinoma separately. Fourthly, given that the radioresistant NSCLC cell line H1299 is p53-null, the p53 pathway genes examined in the study may be regulated through p53-independent mechanisms. Notably, p53 family proteins (p63 and p73) can bind to p53-responsive elements within target genes and contribute to p53 pathway activation. The molecular mechanism by which these p53 pathway-related genes contributes to radiotherapy resistance remains to be elucidated in future studies.

## 5. Conclusions

In this study, a prognostic model based on radiosensitivity- and TP53-signaling-pathway-related genes was constructed for NSCLC. The model demonstrated good predictive efficacy for radiotherapy in a TCGA training set and an exploratory validation set. Immune cell infiltration analysis demonstrated that high-risk NSCLC patients exhibit reduced anti-tumor immune cells infiltration and increased immunosuppressive cells infiltration, and exhibit potential associations with the response to immunotherapy. In vivo and in vitro experiments validate that TP53I3 knockdown enhances NSCLC radiosensitivity through the reduced DNA damage repair. This model holds promise as a tool for risk stratification to guide individualized precision radiotherapy in NSCLC.

## Figures and Tables

**Table 1:** The clinical information of NSCLC patients.

Characteristic	TCGA (n = 105)	In-House (n = 14)
Age (Mean, SD)	63.6 (10.4)	60.5 (7.8)
Gender		
Female	46 (44%)	2 (14%)
Male	59 (56%)	12 (86%)
Clinical stage		
Stage I	24 (23%)	0
Stage II	27 (26%)	4 (29%)
Stage III	45 (43%)	9 (64%)
Stage IV	6 (6%)	1 (7%)
Missing data	3 (3%)	0
Subtype		
Adenocarcinoma	58 (55%)	5 (36%)
Squamous cell carcinoma	47 (45%)	9 (64%)
Smoking history		
Lifelong non-smoker	9 (9%)	1 (7%)
Current smoker	18 (17%)	2 (14%)
Current reformed smoker for >15 years	20 (19%)	4 (29%)
Current reformed smoker for <15 years	50 (48%)	7 (50%)
Missing data	8 (8%)	0

## Data Availability

The TPM-normalized RNA-seq expression data from 14 NSCLC tumor samples have been deposited in the Science Data Bank (ScienceDB, https://doi.org/10.57760/sciencedb.30354, accessed on 22 October 2025), the GSE20549 dataset can be downloaded from the GEO (https://www.ncbi.nlm.nih.gov/geo/, accessed on 5 December 2021), and the TCGA-LUAD and TCGA-LUSC datasets can be downloaded from the UCSC Xena database (https://xenabrowser.net/datapages/, accessed on 5 December 2021).

## Supplementary Materials

The following supporting information can be downloaded at: https://www.mdpi.com/article/10.3390/cancers18030457/s1, original Western blots of Figure 4.

## Abbreviations

The following abbreviations are used in this manuscript:

NSCLC	Non-small cell lung cancer
DEGs	Differentially expressed genes
AUC	Area under the curve
GSEA	Gene set enrichment analysis
TIDE	Tumor Immune Dysfunction and Exclusion
TPM	Transcripts per million
SSD	Source–skin distance
FBS	Fetal bovine serum
PBS	Phosphate-buffered saline
GO	Gene Ontology
KEGG	Kyoto Encyclopedia of Genes and Genomes
CNV	Copy number variation
HRs	Hazard ratios
DDR	DNA damage response
ROS	Reactive oxygen species
NHEJ	Non-homologous end joining
HR	Homologous recombination
TP53I3	Tumor Protein P53 Inducible Protein 3
