# Supplementary figures and images for: A TP53-Pathway-Based Prognostic Signature for Radiotherapy and Functional Validation of TP53I3 in Non-Small-Cell Lung Cancer
Source: Cancers (Basel). 2026 Jan 30;18(3):457. doi: 10.3390/cancers18030457 (PMC12896867; doi:10.3390/cancers18030457)

Figure 4

A

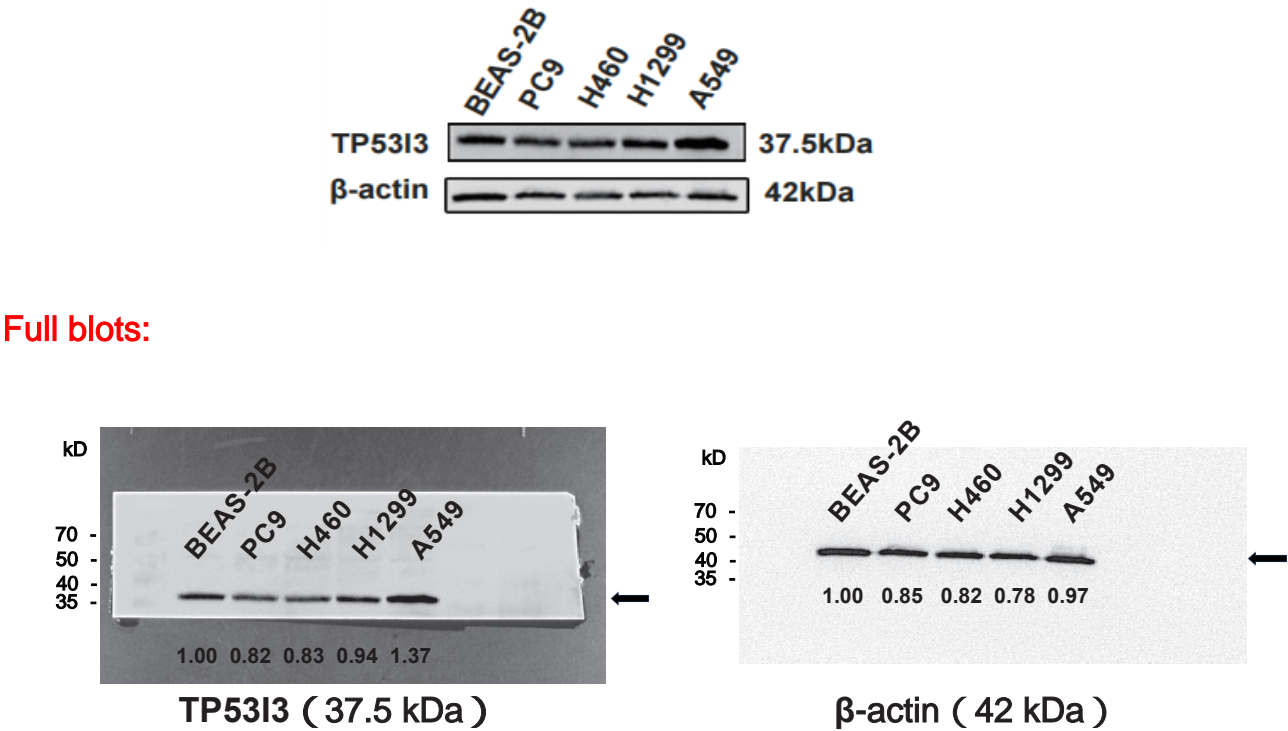

B

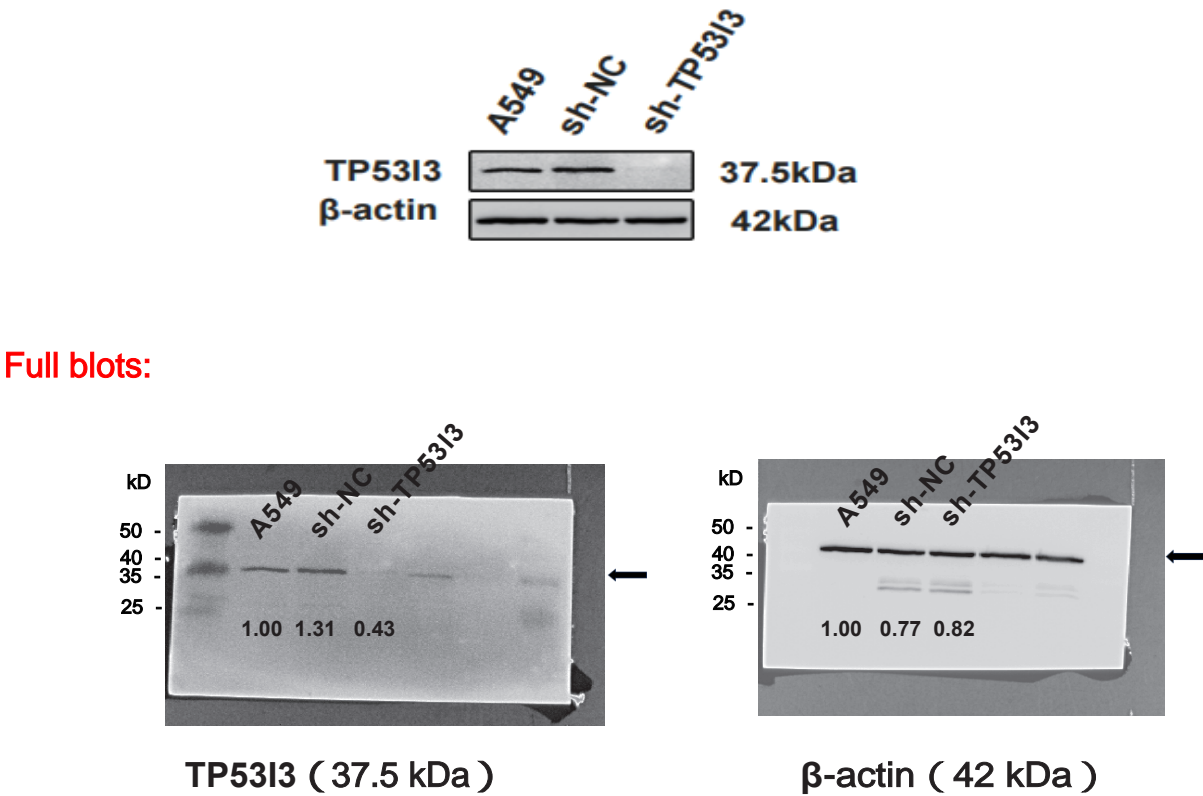

Supplement: Supplementary file 1 [file cancers-18-00457-s001.zip › cancers-4062896-supplementary.pdf]
